# Coexistence of HBsAg and HBsAb in a difficult-to-treat chronic hepatitis B: loss of HBsAg with entecavir plus tenofovir combination

**DOI:** 10.1186/1471-230X-14-94

**Published:** 2014-05-17

**Authors:** Giovanni Galati, Antonio De Vincentis, Umberto Vespasiani-Gentilucci, Paolo Gallo, Donatella Vincenti, Maria Carmela Solmone, Chiara Dell’Unto, Antonio Picardi

**Affiliations:** 1 Clinical Medicine and Hepatology Unit, Campus Bio Medico University of Rome, 200, Alvaro Del Portillo Street, Rome 00128, Italy; 2 National Institute for Infectious Diseases “Lazzaro Spallanzani”, 292 Portuense Street, Rome 00149, Italy

**Keywords:** HBeAg positive chronic hepatitis B, HBsAg, Anti-HBs, Coexistence, Ultra-deep pyro-sequencing, Immunological escape, Nucleos(t)ide analogues, Combination, Entecavir, Tenofovir

## Abstract

**Background:**

Some reports have documented the coexistence of Hepatitis B surfage Antigen (HBsAg) and anti-HBsAg antibodies (HBsAb) in patients with chronic hepatitis B (CHB), often in the absence of amino acid substitutions in the HBsAg sequences of the Hepatitis B Virus (HBV) genome able to explain an immunological escape variant.

HBV genome has a very compact coding organization, with four partially overlapping open reading frames (ORFs). Because the reverse transcriptase region (rt) of HBV polymerase overlaps the HBsAg ORF, it is possible that some mutations in the HBsAg region correspond to mutations in the rt ORF, conferring resistance to current antiviral therapies.

This unique case explores the response to antiviral therapies of a CHB with concurrent HBsAg and HBsAb positivity, and analyse the clinical implications of possible mutations in rt and HBsAg ORFs.

**Case presentation:**

Here we describe the case of a 59 year-old Italian man suffering from Hepatitis B envelope Antigen (HBeAg) positive CHB with concurrent HBsAb positivity. By ultra-deep pyro-sequencing (UDPS) technique, mutations conferring immunological escape or resistance to antiviral therapies were found neither in HBsAg nor in HBV rt ORFs, respectively.

The patient was unsuccessfully treated with interferon, adefovir monotherapy and adefovir plus entecavir combination. Surprisingly, during entecavir plus tenofovir combination, anti-HBe seroconversion and HBsAg loss were observed, while the titer of HBsAb persisted.

**Conclusions:**

Concurrent HBsAg/HBsAb positivity in active CHB is a clinical and virological dilemma. In this setting, there are not consistent data about the response to conventional therapies and the immunological balance between host and virus remains so far unexplained. This is, to our knowledge, the first case described of a CHB with HBsAg/HBsAb positivity, wild type for clinically relevant mutations in HBsAg and rt ORFs, successfully treated with a combination of nucleot(s)ide analogues (NAs).

## Background

Hepatitis B Virus (HBV) can cause a self-limiting acute infection or a chronic hepatitis, depending on the interaction between the host’s immune system and the virus.

Typically, the sign of HBV infection is the presence of Hepatitis B Surface Antigen (HBsAg) in the blood. On the other hand, the appearance of the neutralizing antibodies against HBsAg (HBsAb) usually indicates resolution of infection, both spontaneously and after therapy [1].

In this simple virological scenario, some reports have documented the coexistence of HBsAg and HBsAb in some patients with chronic hepatitis B (CHB), often in the absence of amino acid substitutions in the HBsAg sequence able to explain the escape of HBV from the HBsAb immune control [2,3]. HBV genome has a very compact coding organization, with four partially overlapping open reading frames (ORFs). Because the reverse transcriptase (rt) region of HBV polymerase overlaps the HBsAg ORF, it is possible that mutations in the HBsAg region correspond to mutations in the rt ORF, conferring resistance to nucleos(t)ide analogues (NAs) [4,5]. In addition, due to the quasispecies nature of the HBV genome in each infected individual, some mutations may be present in minor variants of viral population, being not detected by classical population sequencing. The powerful ultra-deep pyro-sequencing (UDPS) approach, based on next generation sequencing (NGS), has been recently used to obtain a complete description of HBV quasispecies, highlighting possible minor populations carrying mutations in the two overlapping ORFs [6].

This case is relevant for clinical virology because explores the response to antiviral therapies of a CHB with concurrent HBsAg and HBsAb positivity, in the absence of clinically relevant mutations in rt and HBsAg ORFs.

## Case presentation

A 59-year-old Italian man was admitted on July 2006 to the Hepatology Unit of the University Hospital “Campus Bio-Medico” of Rome, for investigations concerning CHB. He had not been vaccinated against HBV, he had no known risk factors for contracting viral hepatitis, and all his households were negative for HBsAg. At the time of admission, the virological tests revealed a genotype D hepatitis B envelope antigen (HBeAg) positive CHB with a high viremia (HBV-DNA), mild elevation of ALT (50 IU/ml) and an unexpected low titer of HBsAb (26 mIU/ml, with a protective value above 100 mIU/ml). Anti-hepatitis D and C virus antibodies were negative.

A serological testing performed three years before was diagnostic for HBeAg negative CHB with moderate elevation of ALT (520 IU/ml), medium-low level of HBV-DNA, and absence of HBsAb, suggesting a subsequent seroreversion from HBeAg-negativity/anti-HBe positivity to HBeAg positivity. Till that time, the patient had neither received antiviral drugs nor indication for repeating virological or liver tests.

**Figure 1:**
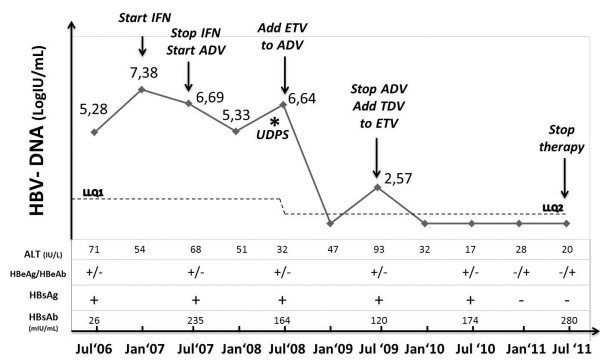
**On-treatment virological and biochemical response.** LLQ1: Lower limit of quantification (Amplicor HBV-Monitor; Roche Diagnostic Systems, Branchburg, NJ); LLQ2: Lower limit of quantification (COBAS TaqMan 48; Roche, Branchburg, NJ); IFN: interferon; ETV: entecavir; ADV: adefovir dipivoxil; TDV: tenofovir. HBsAb quantification (protective value > 100 mIU/ml): Roche Modular Analytics E601 assay.

A liver biopsy was performed showing moderate necroinflammatory activity and bridging fibrosis (Stage 4/6 according to *Ishak’s Score*) [7]. Because the fibrotic evolution, in spite of the genotype D of HBV and the immune-tolerance phase of CHB, antiviral treatment with recombinant IFN-alpha-2b (IntronA®) was began at the dose of 10 MU three times a week. This option was also supported by clinical guidelines at that time, with an expected anti-HBe seroconversion rate of about 37% [8]. During treatment, the patient was monitored by three-monthly blood tests and clinical visits (in Figure 1 six-monthly blood tests are reported).

After six months of interferon treatment, neither a biochemical response nor a virological response were observed, while HBsAb titer raised to 235 mIU/ml (Figure 1). Since consistent data about adefovir dipivoxil (ADV) were already available, the patient was started with ADV (10 mg/day) [9]. Although, after six months, a partial virological response was observed (decrease in HBV DNA of more than 1 log_10_/ml), after twelve months, a virological breakthrough was detected (increase in HBV DNA level of more than 1 log_10_/ml). We were at the beginning of 2007 and the novel carbocyclic analogue of 2' deoxyguanosine Entecavir (ETV) had been just licensed, showing outstanding results [10]. Moreover, due to the persistence of HBsAb with high viremia, mutations in the HBsAg region were investigated. Conventional sequencing and UDPS of the polymerase (pol) region of HBV to identify the presence of mutations in the HBsAg ORF associated to mutations on the overlapping rt ORF, possibly present in minor components of viral quasispecies, were performed. The methods for UDPS and elaboration of sequence data have been previously described [6]. Some mutations in both rt and HBsAg ORFs were detected (with frequency varying from 10 to 96%), but none of these are known to be associated with resistance to current HBV-specific NAs or to be involved in immunological escape (Additional file 1: Table S1).

Based on these results, ETV (0.5 mg/day) was added to ADV and, three months later, HBV-DNA levels became undetectable. On July 2009, after twelve months of ADV and ETV combination therapy, due to an apparent new virological breakthrough (Figure 1), ADV was replaced with the novel nucleotide analogue tenofovir (TDF). After three months of TDF (245 mg/day) plus ETV (0.5 mg/day) combination, virological suppression was achieved. Surprisingly, on January 2011, i.e. six months later, anti-HBe seroconversion and HBsAg loss were observed, while the titer of HBsAb persisted. Anti-HBe seroconversion and HBsAg loss were confirmed after six months, when antiviral agents were discontinued. Unfortunately, the patient died on September 2011 because of a ruptured thoracic aortic aneurysm, so further follow-up visits are not available to verify the sustained off-treatment virological response.

## Conclusions

This case highlights several observations, both in the therapeutic approach and in the virological setting.

In particular, there are some clinical and virological points which deserve consideration: 1) a non-protective HBsAb titer in high-viremic HBV infection; 2) a difficult-to-treat infection, both with IFN therapy and with NAs (ADV and ADV plus ETV); 3) a strong response to second-generation NAs combination (ETV plus TDV), finally with seroconversion to anti-HBe and HBsAg loss.

First, the clinical significance of the co-existence of HBsAg and HBsAb is not well understood, since clinical data are lacking in most studies. A screening of 411 patients with CHB from China revealed a relatively high percentage (4.9%) of detectable serum HBsAb levels, while in a French study a lower prevalence (3.1%) was observed [11,12]. The exposure of patients to HBV infection of different subtypes could not be excluded as a possible explanation of coexistence of HBsAg and HBsAb. The epidemiological importance of such HBV mutants is supported by reports from Taiwan, where the HBV vaccination program was associated with an increased prevalence of HBsAg mutants [13]. Later reports suggested that the presence of HBsAb can drive the selection of HBsAg escape mutants, even if HBV isolates are often without relevant mutations in the coding region of HBsAg, such as in our case [3].

Second, only one case of CHB with a population of HBsAg mutants was described to have seroconversion to anti-HBe and HBsAg clearance after therapy with peg-IFN [14]. Nowadays, it is unknown if these cases are more difficult to cure with standard therapies.

Third, the oral antiviral therapy is able to achieve and maintain virological suppression during long-term use. The most powerful combination of NAs, ETV and TDV, could provide additive or synergistic antiviral activity. Nevertheless, in a study of 379 NAs naïve patients with HBeAg positive and HBeAg negative CHB, the efficacy of ETV was comparable to that of ETV plus TDF combination, in term of HBsAg loss and rate of anti-HBe seroconversion [15]. However, specific data concerning the response to treatment (both with IFN and NAs) of CHB with concurrent HBsAg/HBsAb are lacking, and, from most of clinical studies, results can not even be extrapolated.

Moreover, our patient experienced a seroreversion from HBeAg-negativity/anti-HBe positivity to HBeAg positivity. The appearance and disappearance of HBeAg may be related to the alternating balance of the host immune control over the viral replication, and it could not be excluded that subtle changes in the composition of HBV quasispecies were responsible for the processes of HBeAg seroconversion and seroreversion, as well as for the HBsAg/HBsAb coexistence [16].

The virological findings associated with this case remain so far unexplained. It is possible that genome alterations were present in regions different from those analysed in the study. For this reason, the sequencing of the whole genome would have been probably more informative. Furthermore it is possible that host-associated factors (i.e. immunological competence, innate immunity response, gene polymorphisms associated with poor control of chronic viral infections) may be responsible for the anomalous response pattern observed in this patient.

Finally, we cannot exclude the coexistence of minor mutant strains, not detected by UDPS in serum, that could have been found in the liver or leukocytes.

This is, to our knowledge, the first case described of a CHB with HBsAg/HBsAb positivity, wild type for mutations clinically relevant in HBsAg and rt ORFs, successfully treated with a combination of NAs.

### Consent

Written informed consent was obtained from the patient for publication of this Case report and any accompanying images. A copy of the written consent is available for review by the Editor of this journal.

## Abbreviations

HBsAg: Hepatitis B surfage Antigen; HBsAb: Anti HBsAg antibodies; CHB: Chronic hepatitis B; HBV: Hepatitis B Virus; ORFs: Overlapping open reading frames; Rt: Reverse transcriptase; NAs: Nucleot(s)ide analogues; HBeAg: Hepatitis B envelope Antigen; UDPS: Ultra-deep pyro-sequencing; NGS: Next generation sequencing; IFN: Interferon; ADV: Adefovir dipivoxil; ETV: Entecavir; Pol: Polymerase; TDF: Tenofovir; LLQ: Lower limit of quantification.

## Competing interests

The authors declare that they have no competing interest.

## Authors’ contributions

GG conceived the clinical case and wrote the manuscript; AD helped to draft the manuscript and designed Figure 1 and Additional file 1: Table S1; UV helped to draft the manuscript and revised the written English; PG, DV, MCS, CD and AP helped to draft the manuscript and revised it critically for important intellectual content; DV and MCS performed the ultra-deep pyro-sequencing of the polymerase region of HBV; all authors read and approved the final manuscript.

## Pre-publication history

The pre-publication history for this paper can be accessed here:

http://www.biomedcentral.com/1471-230X/14/94/prepub

## Supplementary Material

Additional file 1 Mutation frequency in rt and HBsAg ORFs by UDPS. Click here for file
