# Supplementary material for: Coexistence of HBsAg and HBsAb in a difficult-to-treat chronic hepatitis B: loss of HBsAg with entecavir plus tenofovir combination
Source: BMC Gastroenterol. 2014 May 17;14:94. doi: 10.1186/1471-230X-14-94 (PMC4031327; doi:10.1186/1471-230X-14-94)
Supplement: Additional file 1 — Mutation frequency in rt and HBsAg ORFs by UDPS. [file 1471-230X-14-94-S1.docx]

| **rt** | **Mutation Frequency (%)** | **HBsAg** | **Mutation Frequency (%)** |
| --- | --- | --- | --- |
| rtG52A | 29 | sG43A | 29 |
| rtP95Q | 15 | sE44P | 29 |
| rtH122F | 10 | sT46A | 33 |
| rtN124H | 10 | sL87R | 15 |
| rtH126R | 10 |  |  |
| rtY221F | 25 |  |  |
| rtL231V | 17 |  |  |
| rtV278A | 23 |  |  |
| rtD283E | 96 |  |  |

**Table S1** Mutation frequency in rt and HBsAg ORFs by UDPS
